# Quantifying the Error of Secondary vs. Distant Primary Calibrations in a Simulated Environment

**DOI:** 10.3389/fgene.2020.00252

**Published:** 2020-03-20

**Authors:** Christopher Lowell Edward Powell, Sydney Waskin, Fabia Ursula Battistuzzi

**Affiliations:** ^1^Department of Biological Sciences, Oakland University, Rochester, MI, United States; ^2^Center for Data Science and Big Data Analytics, Oakland University, Rochester, MI, United States

**Keywords:** molecular clocks, secondary calibrations, simulation, divergence times, timetree, confidence interval

## Abstract

Using calibrations to obtain absolute divergence times is standard practice in molecular clock studies. While the use of primary (e.g., fossil) calibrations is preferred, this approach can be limiting because of their rarity in fast-growing datasets. Thus, alternatives need to be explored, such as the use of secondary (molecularly-derived) calibrations that can anchor a timetree in a larger number of nodes. However, the use of secondary calibrations has been discouraged in the past because of concerns in the error rates of the node estimates they produce with an apparent high precision. Here, we quantify the amount of errors in estimates produced by the use of secondary calibrations relative to true times and primary calibrations placed on distant nodes. We find that, overall, the inaccuracies in estimates based on secondary calibrations are predictable and mirror errors associated with primary calibrations and their confidence intervals. Additionally, we find comparable error rates in estimated times from secondary calibrations and distant primary calibrations, although the precision of estimates derived from distant primary calibrations is roughly twice as good as that of estimates derived from secondary calibrations. This suggests that increasing dataset size to include primary calibrations may produce divergence times that are about as accurate as those from secondary calibrations, albeit with a higher precision. Overall, our results suggest that secondary calibrations may be useful to explore the parameter space of plausible evolutionary scenarios when compared to time estimates obtained with distant primary calibrations.

## Introduction

The use of calibrations to estimate absolute times in a phylogeny is a source of controversy for many reasons; among these are that (i) few are available from independent sources (e.g., fossil record), (ii) their phylogenetic placement can be incorrect, especially in cases of uncertain fossil identification or phylogenetic position, and that (iii) calibration constraints (and the internal distributions between them) are heavily debated, although new methods to estimate probability densities of node ages are being developed (Marjanović and Laurin, 2007, 2008; Ho and Phillips, 2009; Inoue et al., 2010; Sauquet et al., 2012; Sterli et al., 2013; Heath et al., 2014; Hipsley and Müller, 2014; Warnock et al., 2015; dos Reis et al., 2016; Kumar and Hedges, 2016; Didier et al., 2017; Didier and Laurin, 2018; Bromham, 2019; Marshall, 2019). Despite these issues, molecular clock analyses cannot avoid using calibrations if absolute time estimates are the ultimate goal. Alternative approaches that have been explored include the direct use of substitution rates to estimate divergence times, but these present other challenges such as their applicability when, in many cases, these rates are measured from laboratory-grown strains and may not accurately represent rates of strains in the environment (Ho, 2007; Hipsley and Müller, 2014). A recently developed new strategy, instead, suggests the use of horizontal gene transfer events as time-calibrated constraints in a phylogeny (Magnabosco et al., 2018; Wolfe and Fournier, 2018). While this method holds promise, it currently has not been applied widely and the reconstruction of horizontal gene transfer events useful as calibration constraints is a challenging endeavor as it relies on clear gene exchange patterns between groups, one of which should also have primary calibrations. Thus, challenges for these alternative calibration strategies are equally difficult to overcome and, for now, limited improvements have been made. Therefore, the process of assigning time constraints to some nodes in a phylogeny still remains the primary source of information to obtain absolute node times, despite the unbalance between the amount of information (i.e., number of nodes that can be calibrated) available and what would be required to obtain accurate time estimates. This unbalance between availability and need has led researchers to test a variety of alternatives to meet the new needs by increasing calibration sources. These new strategies are especially important in very large phylogenies due to the increase in the ratio of unknown to calibrated nodes and, thus, the potential increase in error propagation of estimates for nodes that are far from a calibration point often caused by rate variation among branches (Britton, 2005; Hug and Roger, 2007; Perie and Doyle, 2012; dos Reis and Yang, 2013; Hipsley and Müller, 2014).

Some examples of alternative strategies to improve the number and quality of available calibrations include (i) the definition of boundaries is either based on the time boundaries of the geologic stratum in which the fossil is found or based on an accurate phylogenetic placement and timing of extinct taxa (Marjanović and Laurin, 2007; Marshall, 2008; Sterli et al., 2013; Warnock et al., 2017) (ii) the selection of representative taxa, which effectively decreases the unknown/calibrated node ratio (Britton, 2005; Perie and Doyle, 2012), and (iii) the use of secondary calibrations (Sauquet et al., 2012; Hipsley and Müller, 2014; Schenk, 2016). In this study we focus on secondary calibrations, which are molecular time estimates obtained from previous molecular clock analyses that were calibrated using independent evidence (primary calibrations). The strongest advantage of using derived (e.g., secondary, tertiary) calibrations is that it effectively provides an infinite source of calibration constraints, only constrained by the number of steps (nodes) a researcher chooses between calibrated and unknown nodes. However, this advantage is dependent on one fundamental question: does the use of derived calibrations result in accurate timetree estimates?

Two past studies have addressed this question with simulation and empirical data analyses, using both Bayesian and maximum-likelihood-based methods (Sauquet et al., 2012; Schenk, 2016). Both found similar outcomes, with secondary calibration estimates being younger than expected and with overly narrow confidence intervals leading to small uncertainties around inaccurate estimates. These results supported previous evidence against the use of secondary calibrations reinforcing the practice of avoiding them, if possible (e.g., Graur and Martin, 2004; Reisz and Müller, 2004; Hug and Roger, 2007; Hipsley and Müller, 2014). However, questions about the performance of secondary calibrations remain. For example, how is the error from primary calibrations compounded into estimates based on secondary calibrations? Can the performance of secondary calibrations be predicted based on uncertainties in the primary calibrations? Is the performance of secondary calibrations worse than that of a small number of primary calibrations that are phylogenetically distant from the nodes of interest (distant primary calibrations)?

To address these questions, we designed a simple simulated scenario with the aim of testing if there are predictable patterns when secondary calibrations are used. For this purpose we used RelTime to estimate times because of its minimal assumption requirements and speed of analyses (Tamura et al., 2012, 2018). In our scenario we used two nested trees that share one overlapping ingroup node that is used as the secondary calibration. In one of the two trees we also selected three nodes to act as primary calibrations. These were used with increasingly larger uncertainties in their boundaries (from 0 to 20% departure from the true, simulated time) and biases (either balanced around the true time or skewed toward younger or older times). Based on the results from previous studies, we expected that estimates based on secondary calibrations would be consistently and precisely underestimated relative to the use of primary calibrations and the true times. Instead, we found that secondary time estimates are generally overestimated by approximately 10% but with low precision (large confidence intervals) and with overall patterns that are clearly predictable. These results suggest that our understanding of the accuracy of secondary calibrations is still incomplete and more comprehensive testing is required to determine their effect if used in empirical datasets.

## Materials and Methods

### Dataset

**FIGURE 1:**
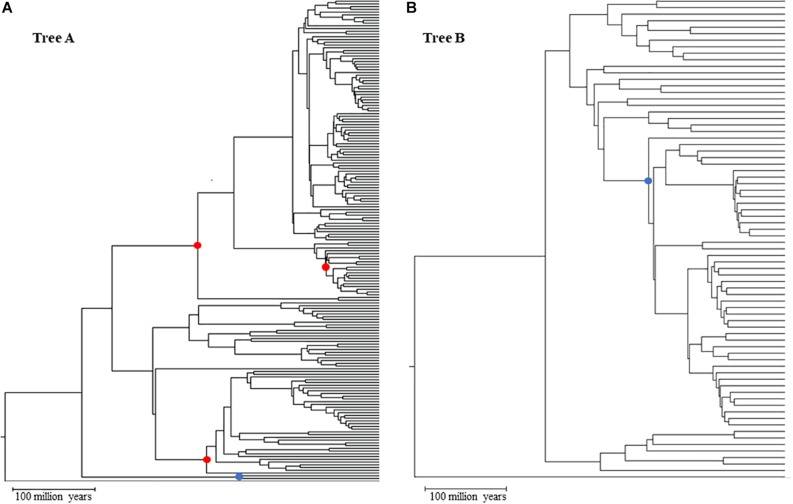
Topologies used in simulated analyses for Tree A **(A)** and Tree B **(B)** red dots primary calibration nodes; blue dot: overlapping node between trees A and B used as secondary calibration in tree **(B)**. Gray branch outgroup.

We started from a main tree of 248 species represented in a tree of life (Hedges and Kumar, 2009). This main tree was split into two subtrees (Figure 1), tree A (173 species) and tree B (71 species), that represent two clades and maximize the size of the dataset in each tree (see Supplementary Data Sheet S5 for NWK formatted tree files). We then added to these clades two shared lineages which were arbitrarily chosen and an outgroup. This setup created two nested phylogenies that were used to test hypotheses on the performance of the calibrations. To simulate multiple genes, we used a set of 446 empirical parameters (e.g., length, GC content, initial evolutionary rate) and altered the main timetree according to an autocorrelated model (ν = 1) that resulted in estimated rates of up to ± 25% of the mean rate (Thorne and Kishino, 2002; Rosenberg and Kumar, 2003) (Supplementary Presentation S1 and Supplementary Figure S1). This effectively created 446 phylogenies with different branch lengths but same topology. These parameters were given to SeqGen to simulate genes under a Hasegawa-Kishino-Yano (HKY) model (Rambaut and Grassly, 1997). Ten random sets of individual genes were then concatenated to reach a length of at least 30,000 sites (30,029–30,725). In addition, we also created one concatenation with all genes (approximately 604,000 sites) and two concatenations of half the number of genes (223 genes per concatenation) with lengths of 273,812 and 330,187. Each of these concatenations were used independently in downstream analyses. Patterns between the 30k, half, and full concatenations were similar. Therefore, we discuss results from the 30k concatenations because they allow us to evaluate the variance of estimates among datasets. For primary calibrations, three nodes from tree A were chosen: a relatively shallow node at 63.9 million years ago (mya), and two that were deeper in the tree but in two different clades (209.4 and 220.2 mya). The overlapping node between tree A and B has an intermediate depth (167 mya) within tree A and is centrally placed within the topology of tree B (Figure 1). These primary and secondary calibrations were chosen to minimize the effect that biased location (e.g., all within one clade) and divergence times (e.g., all young nodes) may have on the accuracy of estimations.

### Time Estimation

Time estimates for each concatenation were calculated using RelTime as implemented in the command-line version of MEGA X (Kumar et al., 2018). Each analysis was run on the Michigan State University HPCC-ICER cluster using a HKY model, uniform rates among sites, all sites, a maximum likelihood estimator, and local clocks. Our goal was to explore the accuracy of time estimates for the nodes in tree B when different types of calibration were used. Thus, we used three combinations of calibrations, all with minimum and maximum constraints: tree A with three primary calibrations (Figure 1, red dots) + tree B with one secondary calibration (this is the overlapping node between tree A and B; Figure 1, blue dot) [B_secondary]; tree B with one primary calibration (same node as the previous combination) (Figure 1, blue dot) [B_primary]; tree AB (the combination of trees A and B) with the same three primary calibrations used in tree A (Figure 1, red dots) which are distant from the nodes in tree B [B_distant_primary]. Additionally, each of these combinations was tested for seven different scenarios that were meant to account for increasing uncertainty on primary calibrations: three of these scenarios had increasingly larger uncertainties, from 0 to 20%, but spread evenly around the true time [0 balanced (0B), 10 balanced (10B), and 20 balanced (20B)]; two scenarios had the error skewed toward younger times [10 low (10L) and 20 low (20L)] and two with the error skewed toward older times [10 high (10H) and 20 high (20H)]. This set up allowed us to test if and how the error in primary calibrations propagates to estimates based on secondary calibrations. Using a subset of our dataset, we also compared our results obtained from tree A using RelTime to results obtained from MCMCTree and found the two to be highly correlated (less than 3% different) (Yang, 2007; Rambaut et al., 2018). Additionally, we tested the potential effect of using a non-partitioned concatenation on the accuracy of the RelTime results by re-analyzing one of our concatenations, partitioning it by gene. Also, in this case, the results are comparable (less than 3% difference), suggesting that the use of a single partition is not biasing the results (see Supplementary Material).

### Measures of Accuracy

We assessed the outcomes of our molecular clock analyses with a number of measures. *First*, we measured the percent departure of the estimated times (ET) from the known (simulated) true times (ET accuracy). *Second*, we calculated the frequency of confidence intervals (CIs) that included the true times (TT) (CI accuracy). *Third*, we calculated the range of each CI normalized to the depth of the node [(maximum–minimum boundary)/TT] (CI precision). *Fourth*, we measured the distance of each CI boundary to the true time [| (CI boundary – TT)/TT] (CI skewness). We applied the latter measure only to the overlapping node between tree A and B to evaluate the effect of skewness on estimates based on secondary calibrations. Each of these measures was applied to all analyses. We report the averages across the 10 concatenations with ± 1 standard deviation in parenthesis because no significant difference was detected among individual concatenations (see Supplementary Data Sheets S1–S4 for concatenation specific estimates).

## Results

Generally, secondary calibrations are used when primary calibrations are not available or are believed to be too few to provide accurate estimates on nodes that are distantly related. While many studies have used this approach as a last-resort method, a systematic evaluation of their performance might open up the use of secondary calibration to more (and larger) phylogenies, thus expanding the applicability of molecular clocks to complex datasets. To investigate and quantify the amount of error associated with secondary calibration, we used a simulated approach by creating two nested trees (A and B) in which one node estimate from primary calibrations in A is used as a secondary calibration in B. We then quantified the accuracy of estimated times (ET) vs. simulated true times (TT) and the properties of the confidence intervals (CIs) in both trees relative to the type of calibration used. Each analysis was repeated for a series of scenarios with variable levels of uncertainty in the primary calibrations to investigate how estimates derived from secondary calibrations may be affected (see section “Materials and Methods”).

In practice, we simulated 100s of nucleotide alignments with a variety of empirically derived parameters (length, initial evolutionary rate, transition/transversion ratio, GC content; Supplementary Figure S1) for a phylogeny of 248 lineages that was split into two nested trees (A with 176 species and B with 74 species). We used variable numbers of genes in concatenation to obtain the alignments used to estimate divergence times. On three nodes in A, we applied primary calibrations with varying levels of uncertainty around their true time (see Table 1). Then, the CI of the molecular time estimate for the common node between A and B was used as secondary calibration for tree B.

**TABLE 1:** Scenarios of varying uncertainty around the true time (TT) for primary calibration boundaries.

Scenario	Calibration boundaries	
	Minimum time	Maximum time
0B	−1 my	+1 my
10B	−5% of TT	+5% of TT
20B	−10% of TT	+10% of TT
10L	−10% of TT	+1 my
10H	−1 my	+ 10% of TT
20L	−20% of TT	+1 my
20H	−1 my	+20% of TT

*Each scenario was applied to all three primary calibrations at once and run independently from the other scenarios. The uncertainty associated with the boundaries varies from a minimum of 0% (±1 million year of the true time) to a maximum of 20%. my: million years.*

Our evaluations of time estimates’ accuracy rely on two basic measures: similarity of the estimated time to the simulated true time (ET accuracy) and the general properties of the CIs (accuracy, precision, skewness). In addition, we also compared estimated times from secondary calibrations to those obtained from B_distant_primary calibrations when the two trees were combined.

### Assessing Accuracy of Primary Calibrations

**FIGURE 2:**
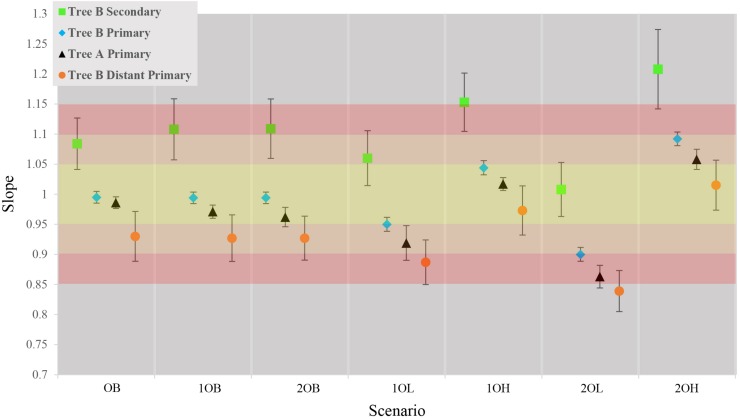
Comparison of average slopes for each of the seven scenarios in the four callibration setting. Gradient of color represents the accuracy of the estimates yellow is ± 5%, Orange ± 10% red = ± 15%. Each data point represents the average slope of the 10 concatensions of true time vs. estimate time with ±1 standard deviation.

The first step was to measure the trends in accuracy of estimated times and CIs from primary calibrations in tree A. On average, the ET differed from TT by approximately 3% with a tendency to underestimate the results (average of slopes = 0.97, *SD* = 0.06) which reflects a generally higher confidence on younger (minimum) calibration boundaries than older (maximum) ones (Figure 2, black triangles; Supplementary Figure S2 and Supplementary Table S1). The observed trends in accuracy were as expected relative to the error associated with calibration boundaries: balanced calibrations (0, 10, and 20B) performed the best, boundaries skewed toward younger times (10 and 20L) produced underestimated times and boundaries skewed toward older times (10 and 20H) produced overestimated times.

On average, the CI ranges included the true time in 87% of the cases with the 0B scenario being the most likely to fail (78% of CIs include the TT) (Table 2). This is expected as narrower calibration boundaries generate narrower CI ranges that are less likely to include the TT in light of the underestimated divergence times. Similarly, in an average of 87% of all the cases the CI included the true time for the node overlapping in trees A and B and, in those cases in which the TT was not included, the minimum boundary was < 5% older than the TT (the maximum boundary was never younger than the TT). In only one scenario, 20H, the difference between TT and minimum boundary was 6.5%, which is expected given the large uncertainty skewed toward deeper times. Moreover, in 93% of the cases the CI of the overlapping node was skewed toward older times which means that, when used as a secondary calibration, this node is expected to behave similarly to the “high” simulated scenarios (see below). Overall, the trends observed from different calibration scenarios in tree A are as expected and provide an accurate basis for the estimation of times using a secondary calibration.

**TABLE 2:** Confidence intervals (CIs) accuracy (proportion of CIs that include the simulated true time) for each of the seven scenarios.

Scenario	CI accuracy			
	Tree A primary	Tree B secondary	Tree B distant primary	Tree B primary
0B	0.78	1.0	0.98	0.91
	(0.1495)	(0.0044)	(0.0222)	(0.0287)
10B	0.87	1.0	0.97	1.0
	(0.1333)	(0.0044)	(0.0306)	(0.0048)
20B	0.95	1.0	0.98	1.00
	(0.0704)	(0.0044)	(0.0181)	(0.0000)
10L	0.86	1.0	0.89	0.99
	(0.0608)	(0.000)	(0.1139)	(0.0000)
10H	0.84	1.0	0.99	0.99
	(0.1590)	(0.0059)	(0.0097)	(0.0125)
20L	0.88	1.00	0.85	1.0
	(0.1223)	(0.0000)	(0.1528)	(0.0042)
20H	0.89	0.99	1.0	0.99
	(0.1658)	(0.0240)	(0.0014)	(0.0097)
Average	0.87	1.0	0.95	0.98
	(0.0525)	(0.0044)	(0.0589)	(0.0313)

*Values shown are averages of all 30k concatenations with 1 standard deviation in parenthesis.*

### Assessing Accuracy of Secondary Calibrations

Using the CI estimated for the overlapping node between tree A and tree B, we obtained secondary divergence times and measured the accuracy of these secondary node ages relative to true times and to estimated times from primary calibrations on distant nodes (we refer to this scenario as B_distant_primary). The first measure is unrealistic in real cases but allows us to quantify the overall error produced by secondary calibrations relative to the true times, while the second measure leads to a quantification of the error introduced by secondary calibrations compared to distant primary ones.

Contrary to previous studies, our results show an average 10% (±6%) overestimation of molecular time estimates against true times with the strongest overestimation in the scenarios with the largest inaccuracy on the maximum boundary (10, 20H) (Figure 2, green squares; Supplementary Figure S3 and Supplementary Table S1). This is predicted from the CI boundaries of the secondary calibrations that are most strongly skewed toward older times in the “high” scenarios. Interestingly, the amount of inaccuracy produced by secondary calibrations is comparable to the average 7% (±6%) departure of the estimates from the true times produced by distant primary calibrations, although in these cases the node ages are generally underestimated (Figure 2, orange circles; Supplementary Figures S4, S5 and Supplementary Table S1).

Despite the overestimation produced by the secondary calibrations, > 99% of CIs include the true time (Table 2). The high probability of the true time being included in the CI of each node is due to the large CI range estimates (78–95% of the true time) (Table 3). This is approximately double the size of the CIs obtained from distant primary calibrations (42–53% of the true time) and from primary calibrations. The larger size of the CIs when secondary calibrations are used reflects the larger uncertainty in the calibrating range. Indeed, while the primary calibrations were allowed to have at most a 20% uncertainty, the CIs of the node used as secondary calibration have, on average, double that amount, thus producing twofold larger CIs in the estimated nodes.

**TABLE 3:** Confidence interval (CI) precision relative to the simulated true time.

Scenario	CI Precision			
	Tree A primary	Tree B secondary	Tree B distant primary	Tree B primary
0B	0.29	0.78	0.45	0.27
	(0.0507)	(0.1409)	(0.1420)	(0.0548)
10B	0.32	0.78	0.43	0.38
	(0.0606)	(0.1740)	(0.0826)	(0.0516)
20B	0.45	0.89	0.49	0.51
	(0.0614)	(0.1756)	(0.0943)	(0.0474)
10L	0.32	0.78	0.42	0.37
	(0.0543)	(0.1626)	(0.0777)	(0.0487)
10H	0.33	0.82	0.45	0.40
	(0.0655)	(0.1867)	(0.0886)	(0.0540)
20L	0.44	0.82	0.48	0.50
	(0.0567)	(0.1316)	(0.0827)	(0.0353)
20H	0.47	0.95	0.53	0.54
	(0.0670)	(0.1780)	(0.1045)	(0.0525)
Average	0.37	0.83	0.46	0.42
	(0.0749)	(0.0065)	(0.0039)	(0.0955)

*Averages of the 30k concatenations with 1 standard deviation are shown in parentheses.*

It is possible that the results obtained in the estimation of node ages for tree B with secondary calibrations could have been driven by anomalies in branch lengths and evolutionary rates specific to this phylogeny rather than the type of calibration used. To identify these potentially confounding factors we first applied a primary calibration on the same node that was used as secondary. If the location and branch lengths associated with the calibration node were biasing the results, the accuracy of estimated times should have been lower even with primary calibrations. Instead, we observed similar accuracy and precision to what was obtained with three calibrations in tree A [Figure 2, blue diamonds; Supplementary Figure S6 and Supplementary Table S1; ET on average within 5% of TT, >98% of CIs include the TT (Table 2), and the CI precision is approximately 40% (Table 3)]. Then, we compared the relative times (before calibrations are applied) of nodes represented only in tree B, only in tree A, and in the combined tree AB. If trees A and B differed substantially in the relative rates of their branches, we would expect that the relative times would not be comparable to those obtained with the combined tree AB. Again, we found the opposite result, which suggests that evolutionary rates do not differ significantly between trees (Supplementary Figure S7). These results show that the estimated times obtained are driven primarily by the choice of calibration and that, therefore, they are a valid measure of calibration performance.

These results show predictable trends for the estimates of secondary calibrations that closely mirror the uncertainty of the primary calibrations. Additionally, they show that, at least in this simulated scenario, the absolute accuracy of using a secondary calibration is similar to that of using distant primary calibrations, although the precision is approximately half.

## Discussion

Despite the potentially broad applications of secondary calibrations, their use has been hindered by concerns over: (i) the process of implementation of time uncertainties from primary calibrations, (ii) the predictability of their performance, and (iii) their overall accuracy and precision. A few studies in the past 15 years have evaluated these three points and generally agreed that secondary calibrations produce systematically biased but precise estimates, effectively attributing to secondary calibrations the worst kind of error: wrongly precise (Graur and Martin, 2004; Hug and Roger, 2007; Sauquet et al., 2012; Hipsley and Müller, 2014; Schenk, 2016). However, some key aspects of secondary calibration assessment are still missing, such as a systematic analysis of how errors in primary calibrations are compounded in the estimates from secondary calibrations and how secondary vs. primary but distant calibrations perform relative to true (simulated) times. Understanding these key aspects would allow us to determine if, and under which conditions, secondary calibrations might produce informative results.

However, because absolute time estimates are the result of the entanglement of evolutionary rates, branch lengths, and calibrations, another fundamental property of a molecular clock assessment analysis is being able to identify the source of observed errors and, if possible, predict the behavior of model parameterizations based on specific scenarios. This approach can be difficult in methods, such as Bayesian, that produce estimates based on many interacting priors. Instead, a theoretically more straightforward approach, such as RelTime, allows to analyze each parameter independently and isolate the source or sources of errors.

Using this approach, we applied secondary calibrations to a suite of simulated alignments with the goal of analyzing three aspects: (i) the overall accuracy of secondary time estimates compared to true times, (ii) the relative accuracy of secondary vs. primary time estimates, (iii) the trends in the errors for the secondary time estimates relative to uncertainties in primary calibrations. By using the same substitution model and topology as in the simulations, we limited issues from phylogenetic uncertainty, and by using uniform, flat distributions for the calibrations we minimized the need to account for decreasing probabilities in the tails of non-uniform calibration distributions. Despite this, our study design has some limitations and caveats that should be taken into consideration when interpreting our results. For example, the use of uniform distributions is not common in empirical data. In real data, calibration constraints are considered more likely to be close to the earliest known fossil evidence for the lineage, thus favoring the use of lognormal, normal, or exponential distributions that are expected to weigh estimates toward younger times (Hedges and Kumar, 2003; Ho and Duchêne, 2014; Ware and Barden, 2016; Didier and Laurin, 2018). Given our overestimated times in the simulations, using lognormal or exponential distributions would likely improve the performance of secondary calibrations. Thus, our use of uniform distributions was more conservative (more likely to highlight estimation biases produced by secondary calibrations). Second, all our primary calibrations have minimum and maximum constraints. Molecular clock methods are known to perform better when both boundaries are provided but this is often not possible in empirical data analysis (Marjanović and Laurin, 2007; Parham et al., 2012; Warnock et al., 2012, 2017). Because one of our goals was to evaluate error propagation from primary to secondary calibrations, providing min-max boundaries allowed us to simulate the exact amount of uncertainties in calibrations and, thus, to track their effect on derived time estimates. The predictable correlation between errors in primary and secondary calibrations could be used to investigate the effect of removing one boundary on a primary node.

The measures we used to determine the effects of the use of secondary calibrations are the typical ones of molecular clock assessment studies: the similarity of true times and estimated times, the frequency of CIs that include the true time, and the precision of CI (their range relative to the age of the node). In addition to these, we also considered the relative error (TT vs. ET) and CI precision of secondary calibrations vs. distant primary calibrations and the predictability of estimates based on secondary calibrations based on the simulated scenario. Surprisingly, our results are opposite to those found by two recent studies: our results show that estimates based on secondary calibrations are, in general, overestimated (by approximately 10%) with poor precision (large CIs). While these results are far from optimal, they show that our understanding of estimates based on secondary calibrations is still incomplete and that their dismissal might be premature. Perhaps more interestingly, we also found that the magnitude of the error in estimates based on secondary calibrations is approximately the same as that produced by the use of distant primary calibrations but in the opposite direction (secondary calibrations overestimate, distant primary ones underestimate). This result is significant because one of the strategies commonly adopted to avoid using secondary calibrations is to increase the dataset size to obtain one or more primary calibrations (Perie and Doyle, 2012). These will inevitably be far away, in the phylogenetic sense, from the nodes of interest, potentially leading to errors, as we see in our simulations. However, an advantage of using distant primary calibrations would be two-fold higher precision (narrower CI ranges) that does not come at the expense of a lower probability of including the true time. It should be noted that in our simulated scenario primary calibrations are given with minimum and maximum boundaries which are expected to increase accuracy and precision. It is possible that the precision of distant primary calibrations would be negatively affected in empirical analyses that do not use maximum constraints (Marjanović and Laurin, 2007, 2008; Marshall, 2019).

A deeper analysis of estimates from proximal primary, distant primary, and secondary calibrations can explain the trends observed. First, the large CI ranges from secondary calibrations are caused by the large uncertainty in the boundaries derived from the primary calibrations. Indeed, these boundaries include a 30–40% error, which is almost double the maximum amount of error assigned to primary calibrations. Therefore, these results suggest that estimates based on secondary calibrations incorporate the error present in the primary estimate in addition to their own. Second, the directionality of the secondary estimated errors (over- or underestimation) is also clearly dependent on the skewness of the primary calibration boundaries. For example, in our simulated cases, we saw that CIs for the overlapping node were almost always skewed toward older times by approximately 30%, driving the observed overestimation. Thus, careful choice of accurate primary calibrations is key when secondary calibrations are to be used. Unfortunately, errors associated with primary calibrations are often unknown in empirical data (but see Didier and Laurin, 2018); but knowing that these errors are included in the estimates based on secondary calibrations with predictable trends makes it possible to test the plausibility of different evolutionary hypotheses based on what is known of the primary calibrations.

A question that remains open is why our results differ so strikingly from those of previous studies. While additional analyses will be necessary to provide an answer, a few hypotheses are possible: first, previous studies used primarily Bayesian methods that are known to depend strongly on priors (dos Reis and Yang, 2013; dos Reis et al., 2016). It is possible that the priors affected the results; but the magnitude of this effect, if present, is unknown. Second, the two previous studies did not take into consideration the error associated with the primary calibrations. In one case (Schenk, 2016) it was not simulated and in the other (Sauquet et al., 2012) it was not known because an empirical dataset was used. From the analyses of multiple scenarios (from many different studies) that the authors carried out it is obvious that the youngest estimates based on secondary calibrations are obtained when only young proximal primary calibrations are used, which is the same trend we observe in our analyses and in other, earlier, studies (Brochu, 2004a, b).

Overall, our simulation study shows that our current understanding of the performance of secondary calibrations is still incomplete and that their dismissal from implementation in favor of other solutions (e.g., distant primary) might not produce the desired increase in accuracy. Additionally, our results show that the performance of secondary calibrations can be predicted based on the uncertainty of the primary calibrations (in our case the different scenarios). Thus, secondary calibrations can be used as a testing tool for different evolutionary scenarios. Therefore, we suggest that rather than avoiding secondary calibrations, they should be used and compared with distant primary ones to test the limits of the parameter space of plausible evolutionary scenarios of divergence times.

## Data Availability Statement

All datasets generated for this study are included in the article/Supplementary Material. All simulated sequences have been uploaded on Dryad (doi: 10.5061/dryad.1zcrjdfp5).

## Author Contributions

CP, SW, and FB designed the project. CP and SW performed the analyses. CP and FB wrote the manuscript.

## Conflict of Interest

The authors declare that the research was conducted in the absence of any commercial or financial relationships that could be construed as a potential conflict of interest.
