# Supplementary material for: Quantifying the Error of Secondary vs. Distant Primary Calibrations in a Simulated Environment
Source: Front Genet. 2020 Mar 20;11:252. doi: 10.3389/fgene.2020.00252 (PMC7099002; doi:10.3389/fgene.2020.00252)
Supplement: Supplementary file 6 [file Presentation_1.pdf]

Supplementary Material

Table S1. Average slope of estimated times *vs.* simulated true times (ET accuracy) for ten 30K concatenations. The slope was calculated from a best fit linear model with intercept = 0. Values for 1 standard deviation are shown in parentheses.

**Scenario**	**ET Accuracy**			
	**Tree A Primary**	**Tree B Secondary**	**Tree B Distant Primary**	**Tree B Primary**
0B	0.99 (0.0097)	1.08 (0.0427)	0.93 (0.0414)	0.99 (0.0097)
10B	0.97 (0.0110)	1.11 (0.0507)	0.93 (0.0386)	0.99 (0.0097)
20B	0.96 (0.0162)	1.11 (0.0493)	0.93 (0.0365)	0.99 (0.0097)
10L	0.92 (0.0288)	1.06 (0.0457)	0.91 (0.0611)	0.95 (0.0115)
10H	1.02 (0.0106)	1.15 (0.0485)	0.98 (0.0443)	1.04 (0.0117)
20L	0.86 (0.0189)	1.01 (0.0449)	0.87 (0.1019)	0.90 (0.0115)
20H	1.06 (0.0169)	1.21 (0.0660)	1.02 (0.0397)	1.09 (0.0113)
Average	*0.97 (0.06363)*	*1.10 (0.0643)*	*0.94 (0.0480)*	*1.00 (0.0617)*

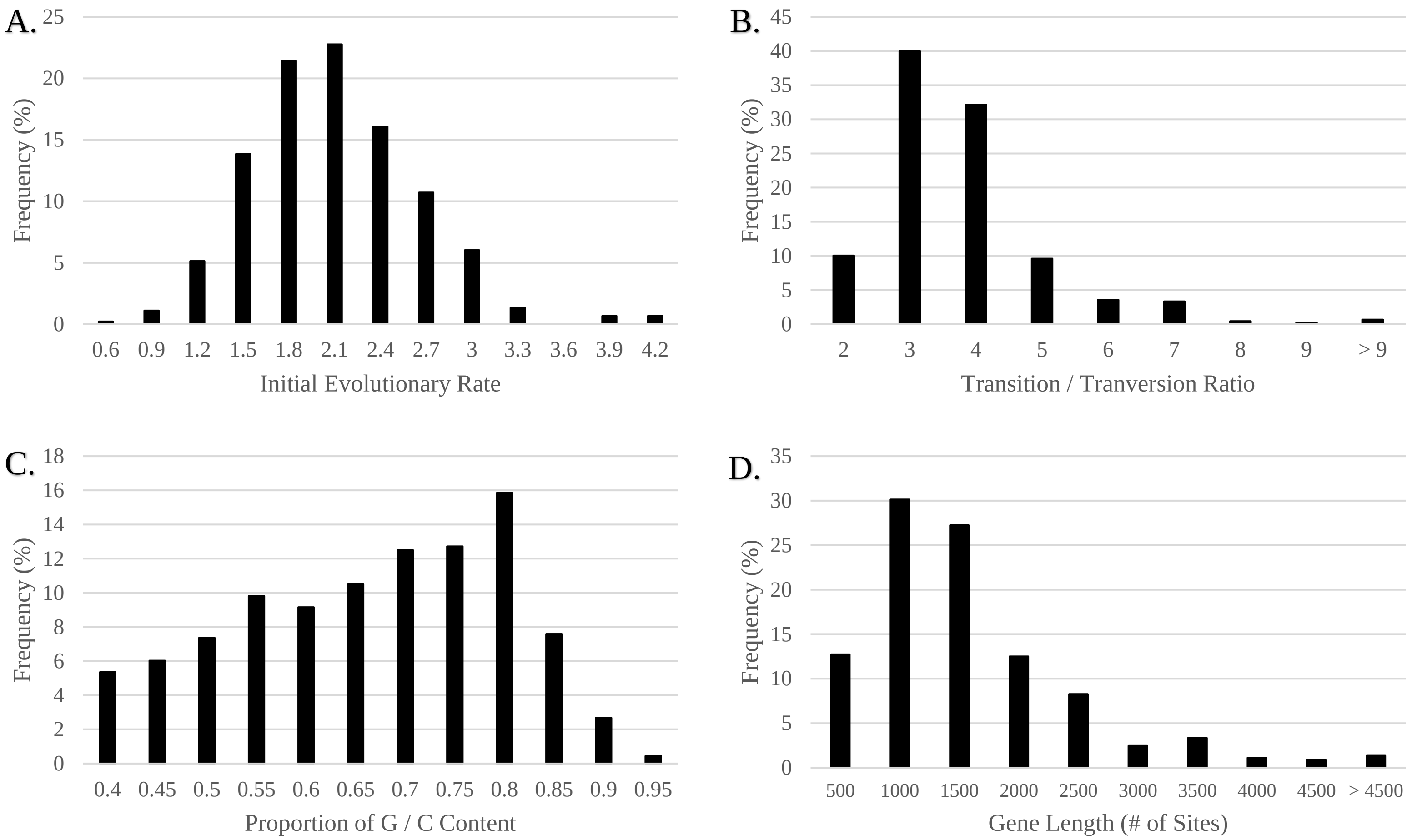

Figure S1. Empirically derived parameters used to simulate alignments A: initial evolutionary rates; B: transversion and transition ratios; C: proportion of guanine and cytosine bases; and D: alignment length of individual genes.

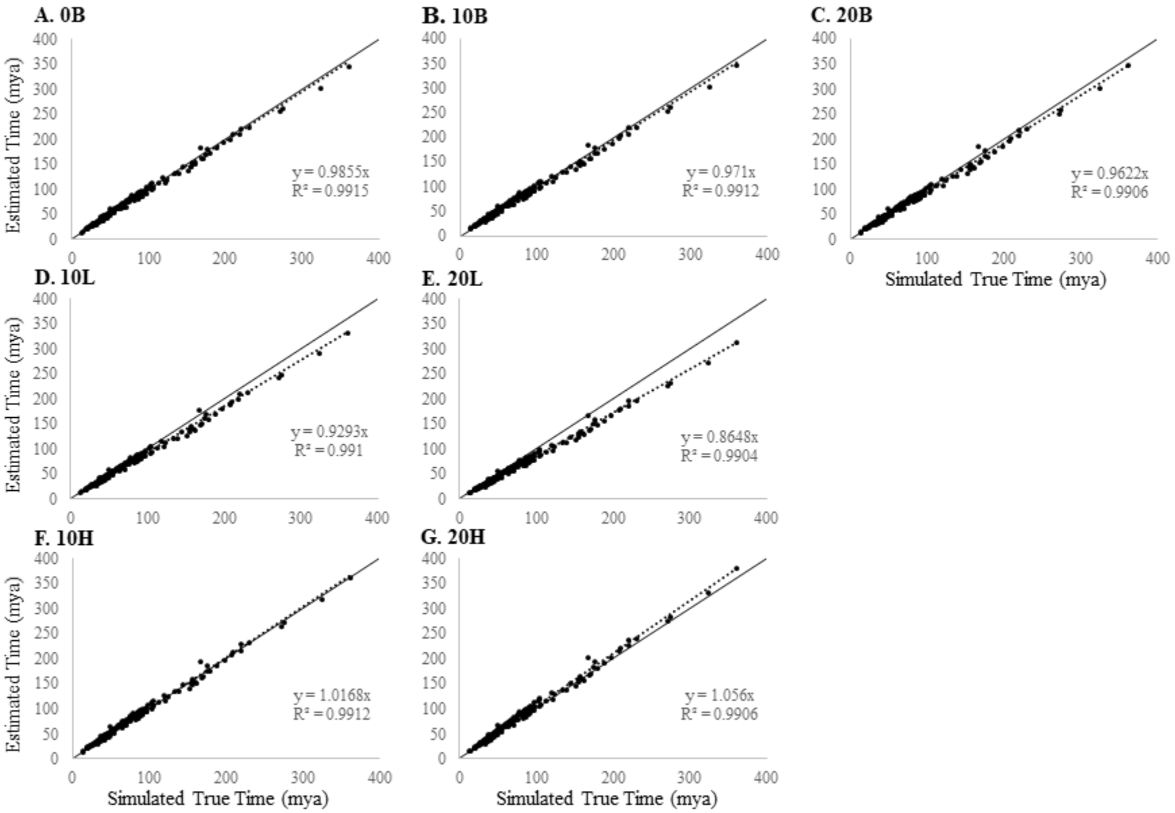

Figure S2. ET accuracy (estimated time *vs.* simulated true time) for tree A with three primary calibrations. The solid line represents a one-to-one match. Equation and R^2 values are shown for each scenario. [Original data in DataSheet1_TreeAPrimary.xlsx]

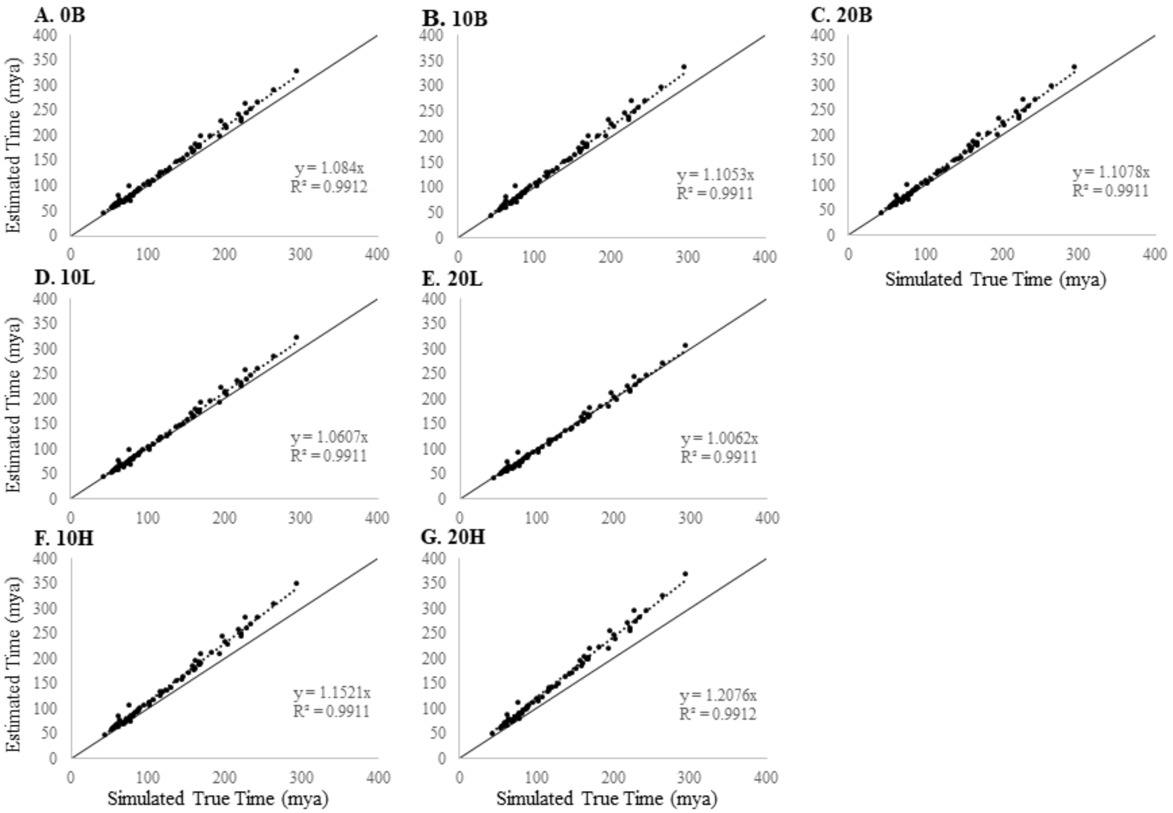

Figure S3. ET accuracy (estimated time *vs.* simulated true time) for tree B with one secondary calibrations. The solid line represents a one-to-one match. Equation and R^2 values are shown for each scenario. [Original data in DataSheet2.TreeBSecondary.xlsx]

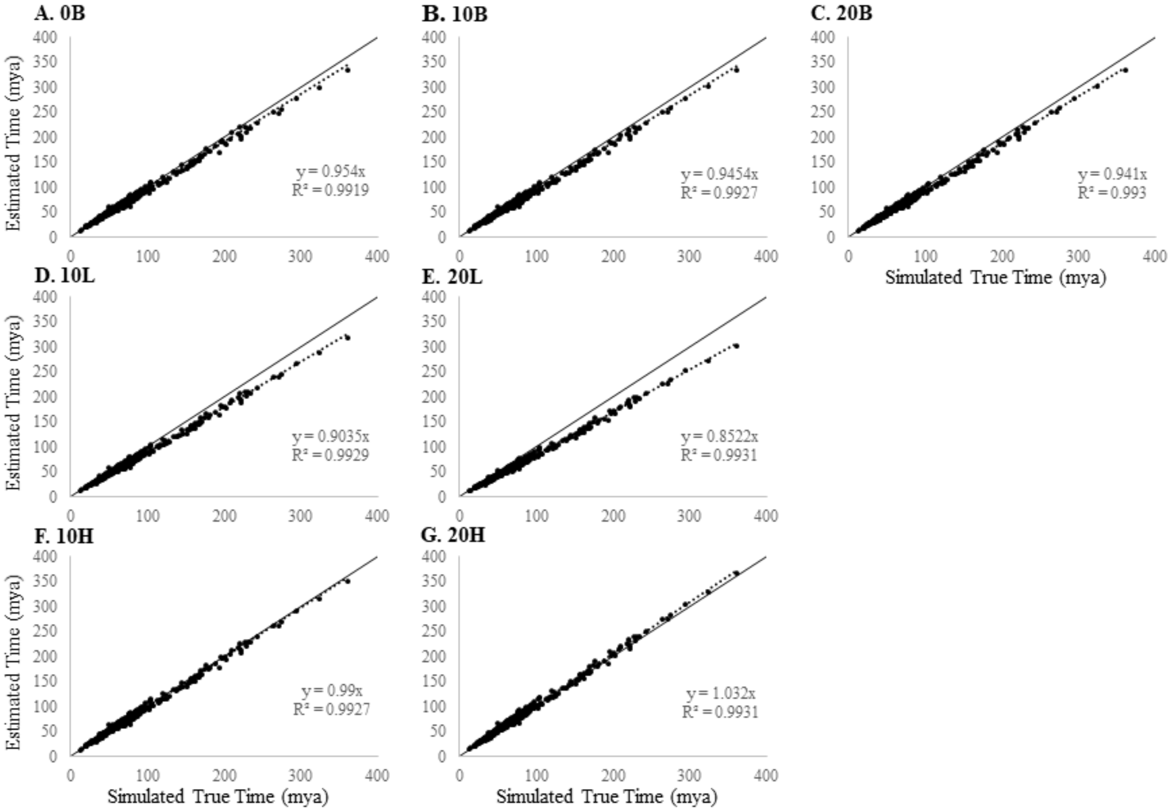

Figure S4. ET accuracy (estimated time *vs.* simulated true time) for tree AB with three distant primary calibrations. The solid line represents a one-to-one match. Equation and R^2 values are shown for each scenario. [Original data in DataSheet3_TreeAB.xlsx]

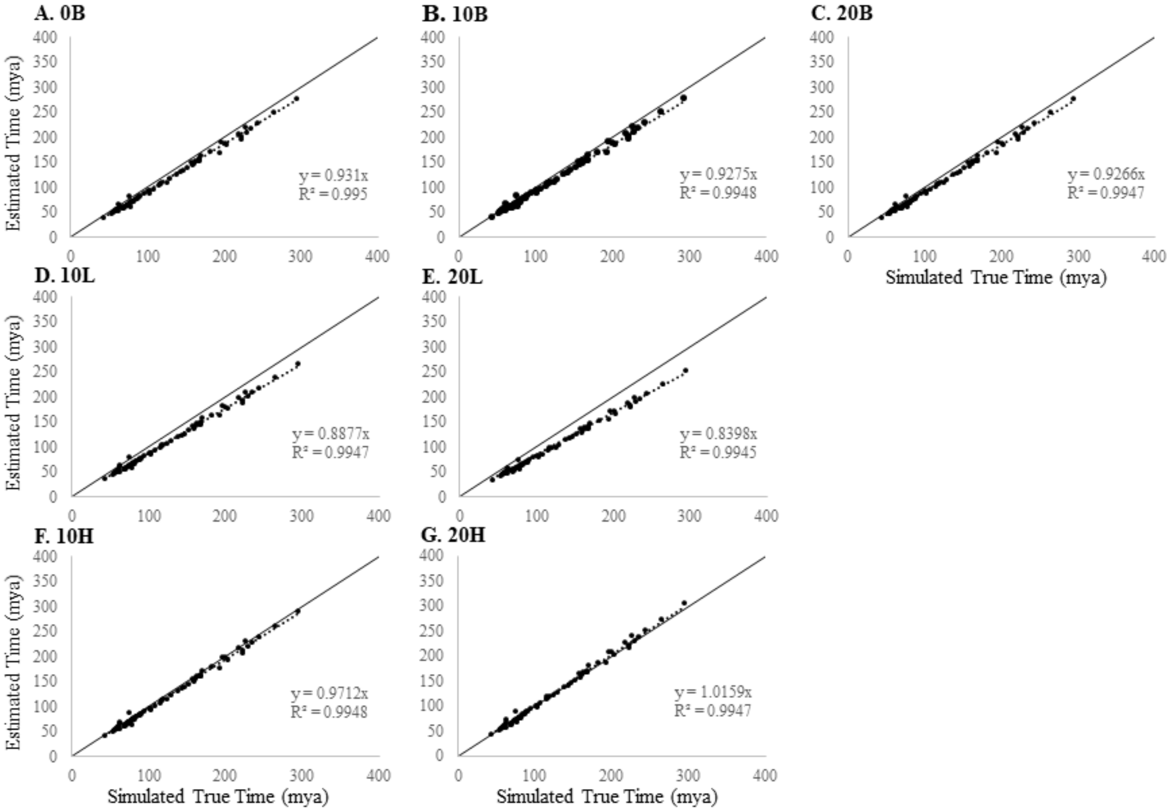

Figure S5. ET accuracy (estimated time *vs.* simulated true time) for tree AB with three distant primary calibrations were only data points for Tree B are plotted. The solid line represents a one-to-one match. Equation and R^2 values are shown for each scenario. [Original data in DataSheet3_TreeAB.xlsx]

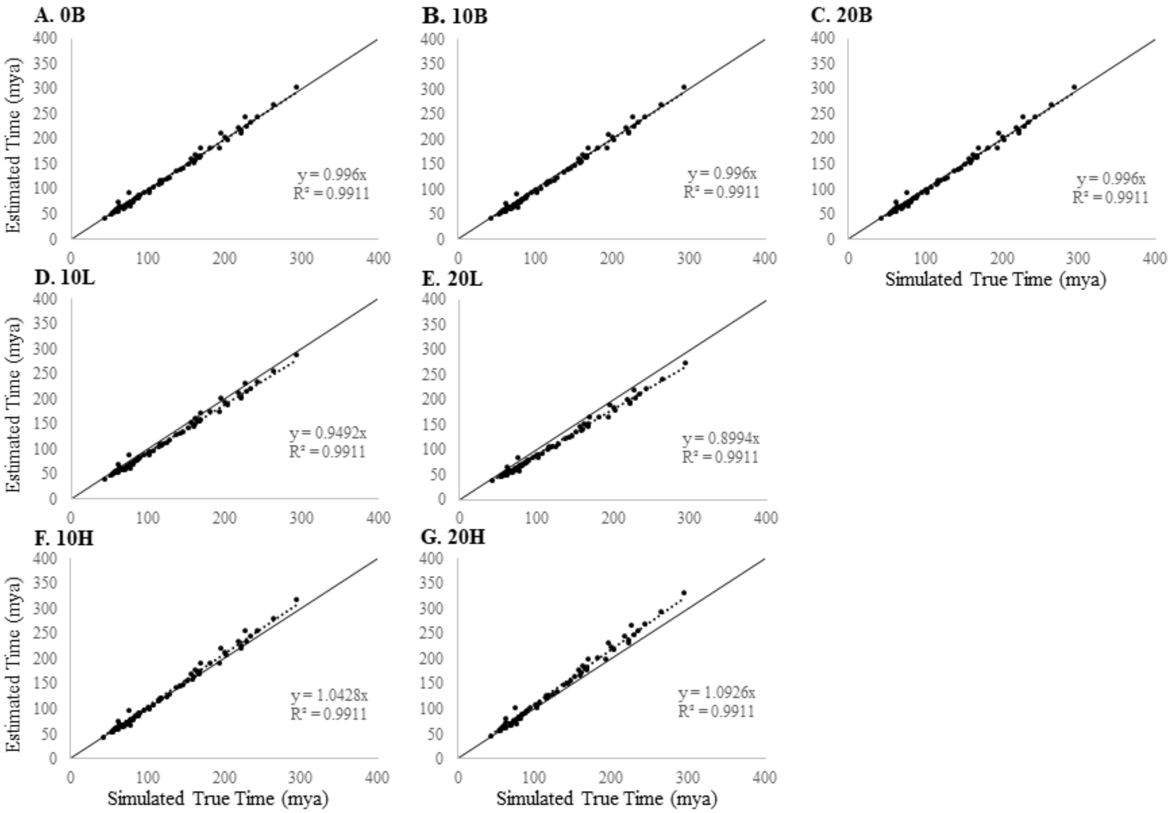

Figure S6. ET accuracy (estimated time *vs.* simulated true time) for Tree B with one primary calibration. The solid line represents a one-to-one match. Equation and R^2 values are shown for each scenario. [Original data in DataSheet4_TreeBPrimary.xlsx]

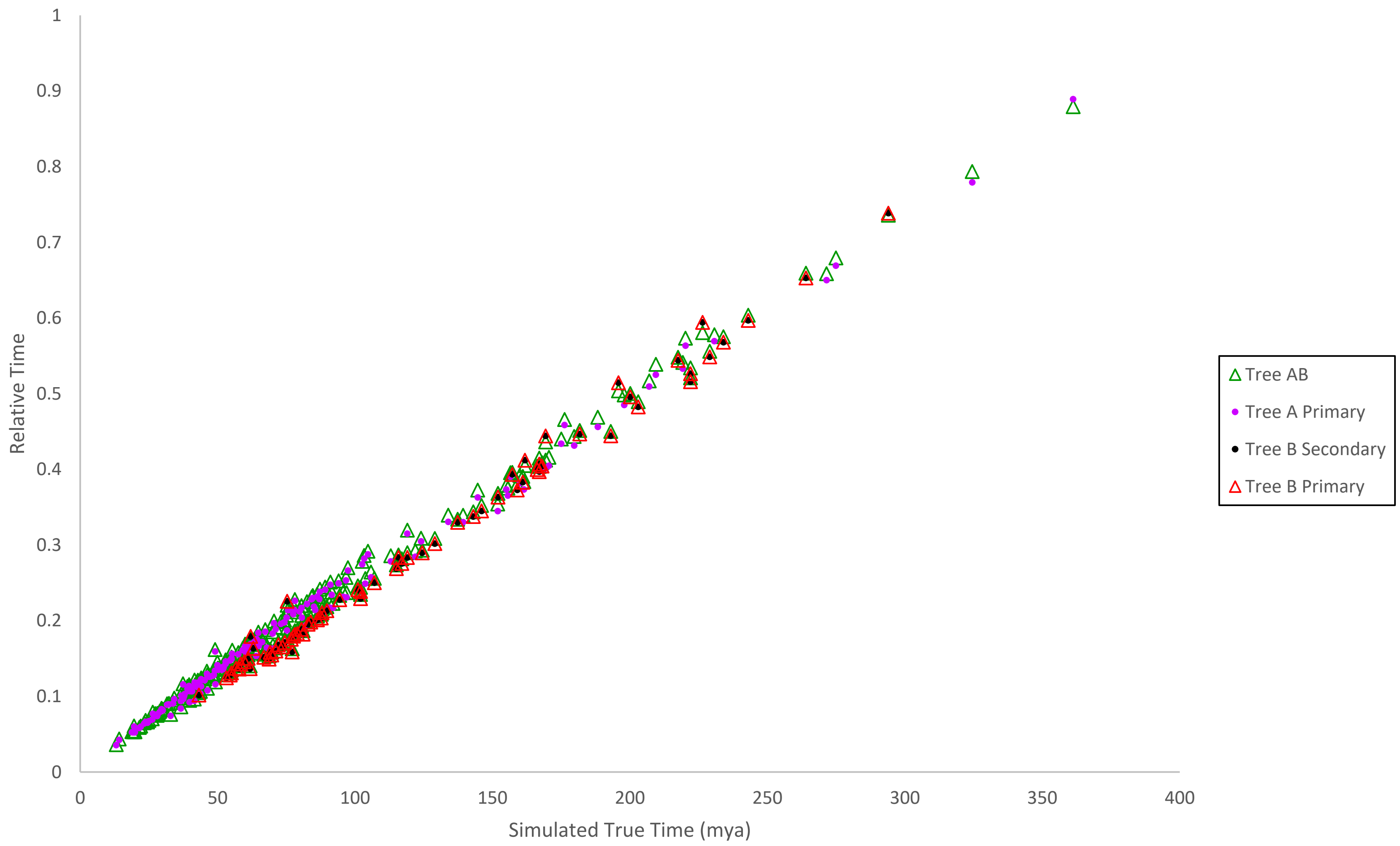

Figure S7. Simulated true times *vs.* relative times from RelTime before calibrations are applied. As expected, tree B relative times before primary or secondary calibrations are applied are the same. Each of the four scenarios contain similar relative time outputs before being calibrated.

Methods

Comparison of MCMCTree and RelTime

Using a subset of our dataset (3 concatenations, two calibration scenarios (0B and 20L) we compared the estimated times obtained using RelTime and MCMCTree (Yang, 2007). MCMCTree was run for 1 million generations, sampling every 100, and with a burnin of 10%. We checked convergence using Tracer v. 1.71 (Rambaut et al., 2018) which showed all ESS values > 100 and the LnL values > 5000. Scatter plots of the RelTime and MCMCTree results show excellent agreement with $R^2 > 0.99$ and slopes between 0.97 and 1.02 (Fig. S8)

Partitioned vs. non-partitioned approach

We also checked if partitioning the data would result in more accurate results. To achieve this, we used RelTime on each gene independently for one of our concatenations (20 genes total), using either 0B or 20L. We then calculated the mean and median of the node ages across each gene and compared these values to the non-partitioned results. We found that both sets of values are within 3% of each, suggesting that a non-partitioned approach is not biasing the results (Fig. S9).

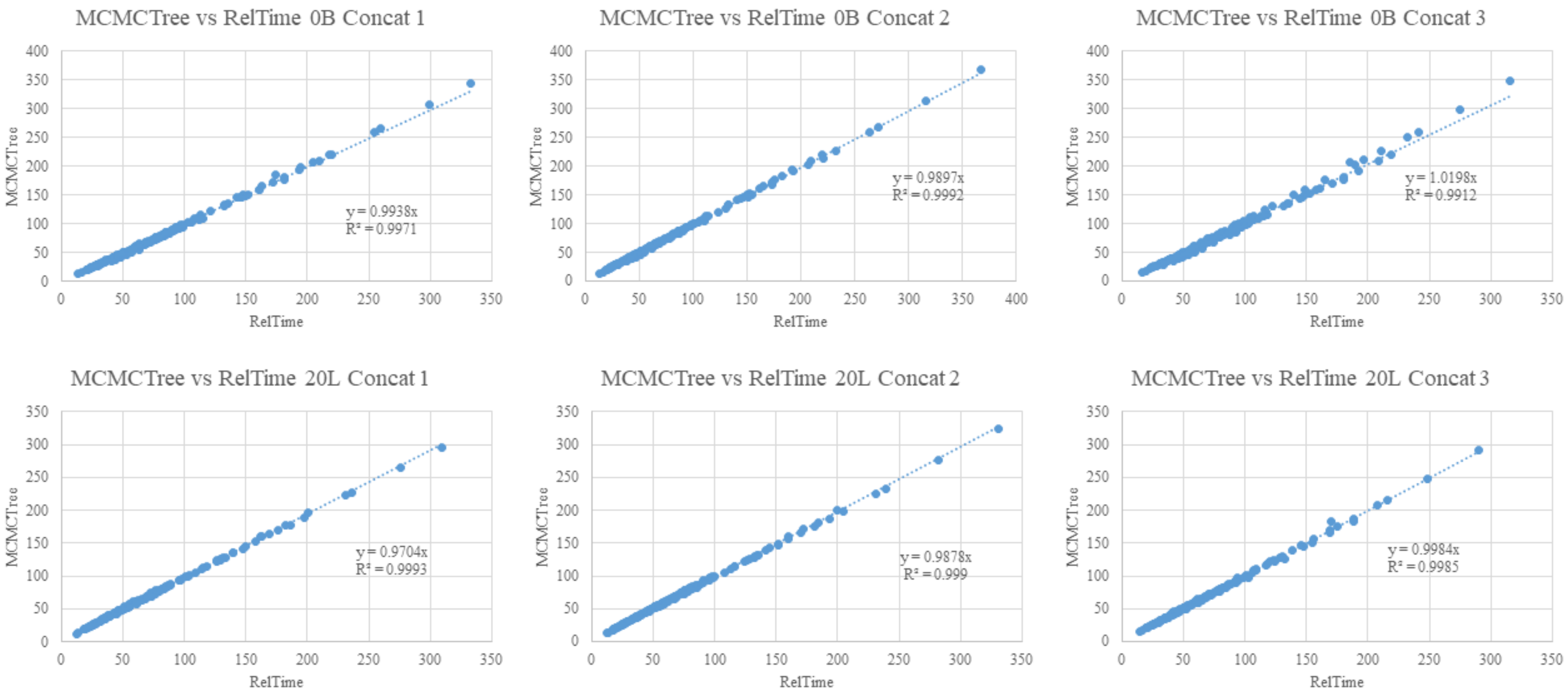

Figure S8. Comparisons of node ages estimated with RelTime and MCMCTree for three concatenations and two calibration scenarios (0B and 20L).

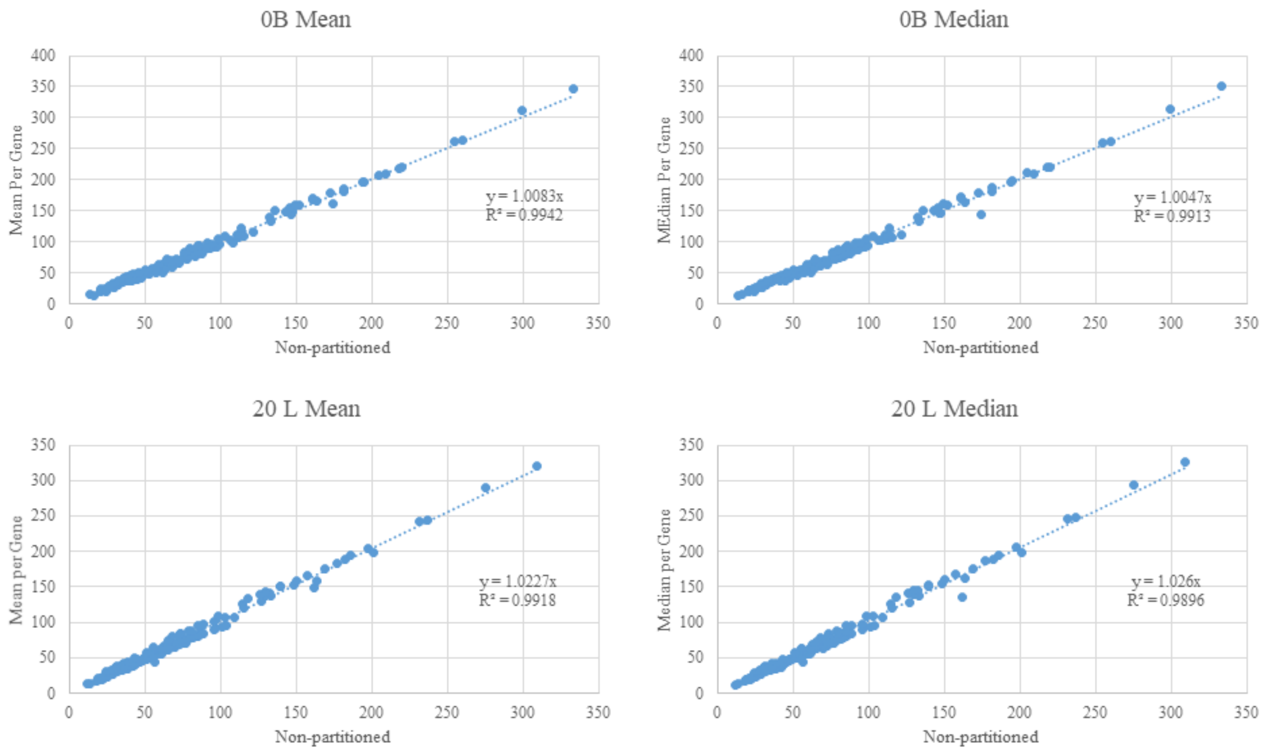

Figure S9. Comparisons of partitioned and non-partitioned time estimates under two calibration scenarios (0B and 20L). Partitioned data were summarized as mean (left plots) and median (right plots).
